# Improving Nursing Staff Attitudes toward Vaccinations through Academic Detailing: The HProImmune Questionnaire as a Tool for Medical Management

**DOI:** 10.3390/ijerph16112006

**Published:** 2019-06-05

**Authors:** Andrea Tamburrano, Claudia Mellucci, Caterina Galletti, Daniela Vitale, Doriana Vallone, Andrea Barbara, Anna Sguera, Maurizio Zega, Gianfranco Damiani, Patrizia Laurenti

**Affiliations:** 1 Institute of Public Health, Università Cattolica del Sacro Cuore, Largo Francesco Vito, 1, 00168 Roma, Italy; claudia.mellucci@gmail.com (C.M.); caterina.galletti@unicatt.it (C.G.); daniela_vitale.dv@libero.it (D.V.); doriana.vallone@guest.policlinicogemelli.it (D.V.); andreabarbara89@hotmail.it (A.B.); gianfranco.damiani@unicatt.it (G.D.); patrizia.laurenti@unicatt.it (P.L.); 2 Fondazione Policlinico Universitario A. Gemelli IRCCS, Largo Agostino Gemelli, 8, 00168 Roma, Italy; anna.sguera@policlinicogemelli.it (A.S.); maurizio.zega@policlinicogemelli.it (M.Z.)

**Keywords:** academic detailing, vaccination, attitude, healthcare workers, nurse, preventive medicine, questionnaire

## Abstract

Vaccinations remain the most effective way of preventing infection, disease, and mortality. Public health institutions consequently recommend vaccines to target groups, including healthcare workers, who are considered to be more at risk of exposure and transmission. The aim of this cross-sectional study is to assess, through the administration of a questionnaire, the nursing staff’s knowledge and attitude towards recommended vaccinations, and to explore the effects of a training course (carried out according to the academic detailing methodology) aimed at increasing operators’ knowledge and outreach on recommended vaccinations among healthcare workers. A total of 85 HCWs (30 nursing coordinators and 55 nurses) completed the questionnaire. Results demonstrate a higher rate of agreement towards vaccinations in nursing staff answers (75%), if compared with results of other studies (62–63%). Statistically significant differences between nursing coordinators and nurses can be found. Regarding vaccination attitudes, nursing coordinators agreed in 86% of the answers on healthcare workers’ vaccination vs 70% of nurses (*p* < 0.001). Considering immunization for influenza, 57% of nursing coordinators vs 18% of nurses reported for vaccination (*p* < 0.001). Educational programs, carried out according to academic detailing methods, could impact on vaccination attitudes and raise awareness about recommended vaccinations among healthcare workers. The questionnaire is a useful tool for investigating nursing staff knowledge and attitudes towards vaccinations, and to implement strategies to promoting vaccinations among healthcare workers.

## 1. Introduction

Vaccination is the first method for preventing the onset and spread of serious infectious diseases and complications. Healthcare workers (HCWs), in addition to being at risk of exposure, are also the cause of the transmission and the spread of infections in the hospital, because of their contact with the patients.

HCWs’ vaccinations are therefore the most important measures for infection control, and are aimed not only at protecting workers from infection, but above all, to avoid flu transmission to vulnerable patients, with considerable benefits for them [1,2].

Even if there is wide evidence that the HCWs’ vaccinations contribute to promoting safety among Healthcare Professionals and patients, in most developed countries, vaccination coverage is low and far from optimal levels [3,4,5]. For this reason, health authorities have made recommendations concerning vaccination [6,7,8]. In a study conducted in a Greek hospital, aimed at evaluating nurses’ knowledge and attitudes concerning recommended vaccinations among HCWs [9], out of 505 nurses who participated in the study, only 19% of them were vaccinated against measles, 19% against mumps, 22% against rubella, 2% against varicella, 4% against hepatitis A, 56% against hepatitis B, and 36% against tetanus–diphtheria. About two third of responders (63%) agreed to mandatory vaccinations for Healthcare Professionals. Similar acceptance rates (68%) were found in a German University Hospital [10], while in an Australian study higher rates were found (91%) [11].

Furthermore, several studies suggested that nursing staff are the most reluctant to vaccinate, compared to other Healthcare Professionals [10,12,13], although it has been widely demonstrated that an adequate flu vaccination coverage is associated with a reduction of absenteeism for workers and mortality for hospital patients [14,15,16].

Aguilar-Diaz et al. [13] demonstrated that the most common reasons for missed vaccinations among HCWs are: fear for adverse events, doubts about efficacy, feelings of not belonging to a high-risk group, and underestimation of disease severity. Seale et al. [11] showed that nurses are more likely not to support the inclusion of vaccinations in hospital policies, compared to other healthcare workers.

An academic detailing strategy was implemented in 1990 as an evidence-based intervention in healthcare, to improve clinical decision making and patient care [17]. Peer education and educational outreach (the basis of academic detailing) have been shown to be effective in different practices and healthcare settings [18]. The need for a change in behaviors, and to improve immunization among HCWs prompted the adoption of this methodology to informative and educational programs in the vaccination field [19,20,21,22,23,24,25]. The purpose was to allow educators and interventionists to promote the effectiveness of recommended vaccinations among HCWs, to ensure patient safety [26].

The aim of this cross-sectional study is to investigate, through the administration of a questionnaire, the prevalence of nursing coordinators and nurse immunizations, and their knowledge, opinions, beliefs, and behaviors toward recommended vaccinations among HCWs.

## 2. Materials and Methods

During the 2017–2018 season, as a part of the flu vaccination campaign, the University Hospital “Fondazione Policlinico A. Gemelli IRCCS” implemented a two-hours training course, aimed at increasing operators’ knowledge and outreach for recommended vaccinations among HCWs, according to AD methodology [17,18,19,21,22,24].

The course was held by a University professor, specialized in the topic of vaccination prevention, and was addressed to all nursing coordinators. During the session, nursing coordinators were invited to answer an anonymous questionnaire. After the course, they were asked to perform educational activities on their nurse staff (according to AD methodology), and to choose a convenience group of nurses to propose the same questionnaire. A total of 85 HCWs completed the questionnaire. All subjects gave their informed consent for inclusion before they administered the questionnaire. The study is compliant with the Local Ethical Committee Standards of the Fondazione Policlinico Universitario Agostino Gemelli IRCCS (Prot. 41409/18, ID: 2263). The study is in accordance with the Helsinki Declaration and EU Regulation 2016/679 (GDPR) concerning the processing of personal data.

### 2.1. The Questionnaire

The survey was taken from the toolkit of the “HProImmune” European Project [27] promoted in Italy by the Istituto Superiore di Sanità [28]. The toolkit was built on a systematic review of past experiences in literature on the HCWs’ vaccination. Vaccination policies and strategies adopted in European countries, vaccination coverages, specific intervention programs, best practices, guidelines, and recommendations were also evaluated for the implementation of the questionnaire [29,30].

The questionnaire, addressed to healthcare professionals, includes a first section of 13 multiple-choice questions about general statements on vaccinations, one multiple-choice question about vaccine recommendations to patients and one open-ended question to note the most well-recommended ones.

Another section of nine multiple-choice questions investigates the health professional’s knowledge about the recommended vaccinations: influenza, varicella, MMR (measles, mumps, rubella), hepatitis A and B, Tdap (tetanus, diphtheria and pertussis) or Td (tetanus, diphtheria) for adults, anti-pneumococcal, anti-meningococcal, and anti-tuberculosis vaccines.

Further sections of 10 multiple-choice questions are about the HCWs’ immunization history.

The last section collects information on personal data: gender, age, qualification, and the number of years of service.

The questionnaire was administered in paper form, collected anonymously, and subsequently transferred to an electronic database.

A group of questions in the first section were classified into three categories to investigate differences between responders:

1. Adverse/side effects of vaccination (questions 5, 6, 11);
2. Inefficacy of vaccination (questions 4, 10);
3. Importance of vaccination (questions 1, 2, 12 and 13).

Finally, we made an overall assessment (questions 1, 2, 4–6, 10–13) on responders’ attitudes towards vaccinations among HCWs.

Answers “in total disagreement” and “in disagreement” were considered “disagreements”, answers “agreements” and “fully agreement” was considered to be “agreement”. “Unsure” responders were also investigated.

### 2.2. Analysis

Descriptive statistics were performed, analyzing the frequency, percentage, mean, and standard deviation.

Section 1 and Section 2 (based on a 5-point Likert scale) were investigated by using the Mann-Whitney U test for unpaired data, to explore the differences in terms of vaccination attitude by qualification, age, and gender.

Other sections were analyzed by using the Yates’s Chi-squared test, to explore differences in terms of immunization, knowledge, opinions, beliefs and behaviors between nursing coordinators and nurses.

The level of significance was set at 0.05. Statistical analyses were conducted with STATA software ver.13.1 (Statacorp, College Station, TX, USA).

## 3. Results

A total of 85 HCWs (30 nursing coordinators and 55 nurses) completed the questionnaire (100% response rate). Their characteristics are shown in Table 1. Moreover, frequency distribution of the answers to the first two sections (based on a 5-point Likert scale) are shown in Table 2.

**Figure 1:**
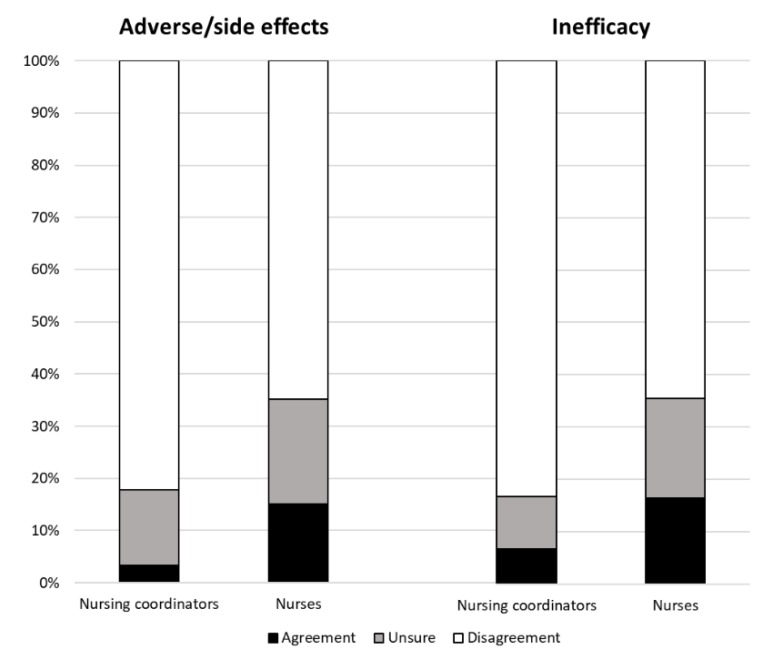
Frequency distribution (percentages) of the answers on the “adverse/side effects” and the “inefficacy” categories by qualification.

Considering the “adverse/side effects of vaccination” among healthcare workers (Figure 1), nursing coordinators agreed in three answers (3%), vs. 25 (15%) of nurses. There were also 13 answers (14%) of unsure nursing coordinators and 33 (20%) of unsure nurses. This difference by qualification was statistically significant (*p* < 0.001).

No statistically significant differences by age and gender were observed.

Regarding the “inefficacy of vaccination” among healthcare workers (Figure 1), nursing coordinators agreed in four answers (7%), vs. 18 16%) of nurses. There were also six answers (10%) of unsure nursing coordinators, and 21 (19%) of unsure nurses. This difference by qualification was statistically significant (*p* < 0.001).

Non-statistically significant differences by age and gender were observed.

**Figure 2:**
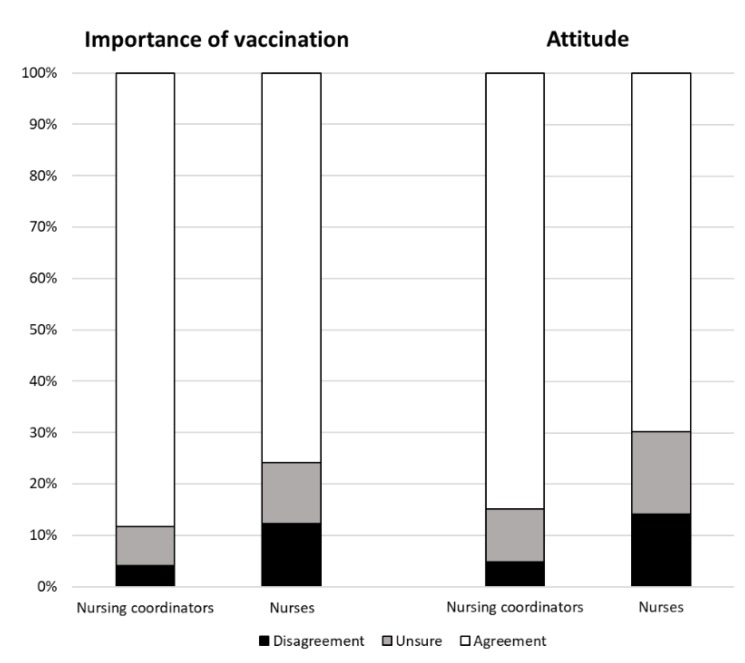
Frequency distribution (percentages) of answers on the “importance” and the “attitude” categories by qualification.

Considering the “importance of vaccination” among healthcare workers (Figure 2), nursing coordinators agreed in 106 answers (89%), compared to 167 (76%) of nurses. The difference by qualification was statistically significant (*p* = 0.018). No differences by age and gender were observed.

Finally, we made an overall assessment considering interviewees’ “attitudes towards vaccination” among healthcare workers (Figure 2). Nursing coordinators agreed on the practice of HCWs’ vaccination in 229 answers (86%), vs. 345 (70%) of nurses. There were also 28 answers (10%) of unsure nursing coordinators, and 80 (16%) of unsure nurses. This difference by qualification was statistically significant (*p* < 0.001). No differences by age and gender were observed.

The frequency distributions of the answers to the other sections of the questionnaire, concerning behaviors, knowledge, immunization, and intention to vaccinate among nursing coordinators and nurses, are shown in the following tables.

To the question “In your clinical practice, do you recommend vaccinations to your patients?” (Table 3), 31 responders (36%) fully recommended vaccinations, 24 (28%) recommended vaccinations only in some cases, and 30 (35%) never recommended vaccinations. No differences by qualification, age, or gender were observed.

To the question “Do you know which of the following vaccinations are recommended for healthcare professionals?” (Table 4), most of the responders identified hepatitis B (78, 92%), influenza (75, 88%), and MMR (66, 78%) as “recommended” vaccinations. A low identification concerned hepatitis A (19, 22%), anti-meningococcal (35, 41%) and anti-pneumococcal (24, 28%). No differences by qualification, age or gender were observed.

To the statement “You get vaccinated for” (Table 5), the most part of responders got vaccinated for hepatitis B (79, 93%), MMR (73, 86%), and varicella (69, 81%). The lowest rate was for flu vaccination (27, 32%). Considering qualification of responders, 17 (57%) nursing coordinators vs. 10 (18%) of nurses reported to get vaccinated for influenza. The difference was statistically significant (*p* < 0.001).

## 4. Discussion

Through this study, we observed a positive attitude by healthcare workers towards vaccinations. In particular, our results demonstrate a higher rate of agreement in nursing staff answers (75%), if compared with the results of other studies: in a Greek hospital [9] 62%, in a German University Hospital [10] 63% of nurses agreed to vaccines. These results confirm the efficacy of the academic detailing methodology applied in the implementation of the training courses, which determined a significant improvement in the nursing staff attitude toward vaccinations.

Only 31 (36%) responders fully recommended vaccinations to their patients. and similar results can be found in a study conducted in 3 hospitals in Jiangsu Province, China [31], where 38% of the nursing staff referred to usually recommend vaccination to the patients.

In addition, the nursing staff knowledge on the recommended vaccinations for the HCWs was extremely variable: from low percentages for hepatitis A (22%) and anti-meningococcal (41%) to high percentages for hepatitis B vaccine (92%). Similarly, in a survey conducted by Maltezou et aI. [9], 90% of HCWs identified hepatitis B as a recommended vaccination, and only 26% for hepatitis A.

Likewise, nursing staff immunization was variable: from a high percentages for hepatitis B vaccine (93%) to low percentages for the flu vaccine (32%). For the influenza vaccine, coverage differences by qualification can be found: 57% of nursing coordinators vs 18% of nurses. Loulergue et al. [32] registered the same HCWs’ coverage for hepatitis B (93%); flu vaccination, instead, had an higher rate (49%). Maltezou et al. [9] reported a lower rate for hepatitis B (56%) and a similar rate for influenza (33%).

The results highlight a high number of “unsure” responders: 10% of nursing coordinators’ and 16% of nurses’ answers respectively. This might be related to general skepticism and poor knowledge toward HCWs’ recommended vaccinations, as reported in a French study [32], where only 22% of nurses agreed to vaccinations.

However, our study highlights some relevant differences among respondents, especially by professional roles. Considering the “importance of vaccination” among healthcare workers, there was a greater awareness within the nursing coordinators’ (89%) rather than the nurses’ (76%) answers. For the “adverse/side effects of vaccination”, in 15% of answers, nurses were more afraid of vaccination than nursing coordinators (3%). Regarding the “inefficacy of vaccination”, nurses were more doubtful (16% of answers) than nursing coordinators (7%). Overall, nursing coordinators had a better “attitude towards vaccinations” than nurses (85% vs. 70% of the answers). In only 5% of their answers, nursing coordinators disagreed on vaccination among health care workers, compared to 14% of nurses.

One of the strategies for increasing HCWs’ compliance with vaccination can be a specific training course that is addressed to the nursing staff, doctors, and other health care workers, according to the academic detailing methodology; lecture days (led by specialized doctors and supported by nurses) might be good interventions to spread the awareness of vaccination, especially among the nursing staff. Significant positive effects could be achieved in terms of changing perceptions and knowledge, especially in the insecure staff. The reduction of the nursing staff hesitancy would allow for the 95% coverage goal set by the World Health Organization to be reached [33]. In this way, optimal coverage levels could be reached in a few years.

## 5. Strengths and Limitations

Convenience sampling represents a first limit of this study. Despite a small number of responders, a large number of questions, which investigate many aspects (behavior, knowledge, immunization), provides a wide representation of the nursing staff attitude to vaccinations among HCWs. A second limit concerns training, which was directly administered to nursing coordinators only (according to the principles of the academic detailing methodology). This could encourage new studies to consider the wide participation of all healthcare professionals, to further informational training initiatives.

## 6. Conclusions

The questionnaire we used was a useful source of monitoring nursing staff beliefs and attitudes about vaccinations. In combination with the trend analysis of annual coverage among HCW, it could help Medical Management to implement strategies to promote vaccination, providing a better understanding of the barriers for the acceptance of vaccination, especially in a large University hospital.

Further studies may be needed to evaluate vaccination attitudes amongst the nursing staff, also investigating the correlation between attitude, nursing staff seniority, and the level of qualification. More evidence will serve a useful part in improving informative and educational programs that are aimed at ensuring patient safety as a main goal of public health, as well as in the hospital setting. Through surveillance, nursing staff are critical for reducing adverse events and negative outcomes that derive from the spread of infectious diseases in the hospital, and this are avoidable with the vaccination of patients and HCWs.

## Figures and Tables

**Table 1:** Frequency distribution (values and row percentages), means and standard deviations of the sample’s characteristics.

Qualification	Length of Service (Mean ± SD)	Female		Male		Total
**Nursing coordinators**	26.6 ± 7.6	24 (80%)		6 (20%)		30
**Nurses**	19.6 ± 9.1	42 (76%)		13 (24%)		55
**Total**	22.2 ± 9.2	66 (78%)		19 (22%)		85
**Age (years)**	20–29	30–39	40–49		50–59	>60
**Nursing coordinators**	0 (0%)	3 (10%)	12 (40%)		13 (43%)	2 (7%)
**Nurses**	7 (13%)	10 (18%)	23 (42%)		12 (22%)	3 (5%)
**Total**	7 (8%)	13 (15%)	35 (41%)		25 (29%)	5 (6%)

**Table 2:** Frequency distribution (values and row percentages) of the answers to Section 1 and Section 2.

Questions	Total Disagreement	Disagreement	Unsure	Agreement	Full Agreement
**I believe that vaccines are important for reducing or eliminating serious illnesses**	0 (0%)	1 (1%)	3 (4%)	32 (38%)	49 (58%)
**I believe that vaccines are useful in certain situations, for example, in developing countries**	2 (2%)	2 (2%)	3 (4%)	44 (52%)	34 (40%)
**I have no opinion on it**	26 (54%)	19 (40%)	2 (4%)	1 (2%)	0 (0%)
**I believe more in the natural immunity gained through the disease than in vaccines**	17 (20%)	30 (35%)	23 (27%)	9 (11%)	6 (7%)
**I do not believe in vaccination: I think it does more harm than good**	38 (45%)	34 (40%)	11 (13%)	2 (2%)	0 (0%)
**I am afraid of side effects**	15 (18%)	30 (35%)	22 (26%)	16 (19%)	2 (2%)
**My religious beliefs are against vaccinations**	63 (76%)	19 (23%)	1 (1%)	0 (0%)	0 (0%)
**I don’t believe to be at risk for infectious diseases**	46 (55%)	23 (28%)	11 (13%)	3 (4%)	0 (0%)
**I’m afraid of getting sick after vaccination**	28 (33%)	30 (36%)	17 (20%)	9 (11%)	0 (0%)
**I believe that vaccines are not effective**	34 (40%)	40 (47%)	4 (5%)	3 (4%)	4 (5%)
**I am wary of vaccination long-term effects on health of vaccinations**	21 (25%)	43 (51%)	13 (15%)	8 (9%)	0 (0%)
**I believe that vaccinations are an indispensable requirement to work in a health care setting**	0 (0%)	11 (13%)	16 (19%)	39 (46%)	19 (22%)
**I believe that vaccinations are a duty because health care workers should represent a model for their patients**	2 (2%)	14 (16%)	13 (15%)	39 (46%)	17 (20%)

**Table 3:** Frequency distributions (values and row percentages) and *p*-values (Yates’s chi-squared test) of the answers to the question “In your clinical practice, do you recommend vaccinations to your patients?”.

Answer	Nursing Coordinators	Nurses	Total	*p*-Value
**Yes**	11 (37%)	20 (36%)	31 (36%)	0.710
**Sometimes**	7 (23%)	17 (31%)	24 (28%)	
**No**	12 (40%)	18 (33%)	30 (35%)	

**Table 4:** Frequency distribution (values and percentages) and *p*-value (Yates’s chi-squared test) of the answers to the question “Do you know which of the following vaccinations are recommended for healthcare professionals?”.

Answer	Nursing Coordinators	Nurses	Total	*p*-Value
**Influenza**	25 (83%)	50 (91%)	75 (88%)	0.494
**Varicella**	15 (50%)	39 (71%)	54 (64%)	0.093
**MMR**	22 (73%)	44 (80%)	66 (78%)	0.665
**Hepatitis B**	28 (93%)	50 (91%)	78 (92%)	0.981
**Hepatitis A**	8 (27%)	11 (20%)	19 (22%)	0.665
**Tdap or Td**	15 (50%)	29 (53%)	44 (52%)	0.989
**Anti-pneumococcal**	11 (37%)	13 (24%)	24 (28%)	0.308
**Anti-meningococcal (tetravalent)**	12 (40%)	23 (42%)	35 (41%)	0.946
**BCG (anti-Tuberculosis)**	22 (73%)	42 (76%)	64 (75%)	0.963

MMR: measles-mumps-rubella; Tdap: tetanus, diphteria and pertussis; Td: tetanus, diphteria; BCG: Bacillus Calmette-Guérin.

**Table 5:** Frequency distribution (values and percentages) and *p*-value (Yates’s chi-squared test) of the answers to the statement “You get vaccinated for”.

Answer	Nursing Coordinators	Nurses	Total	*p*-Value
**Influenza**	17 (57%)	10 (18%)	27 (32%)	<0.001
**Varicella**	24 (80%)	45 (82%)	69 (81%)	0.874
**MMR**	27 (90%)	46 (84%)	73 (86%)	0.431
**Hepatitis B**	28 (93%)	51 (93%)	79 (93%)	0.357
**Tdap or Td**	20 (67%)	34 (62%)	54 (64%)	0.453

MMR: measles-mumps-rubella; Tdap: tetanus, diphteria and pertussis; Td: tetanus, diphteria.
